# Effect of troglitazone on tumor growth and pulmonary metastasis development of the mouse osteosarcoma cell line LM8

**DOI:** 10.1186/1471-2407-10-51

**Published:** 2010-02-22

**Authors:** Junichi Aizawa, Kenshi Sakayama, Setsuya Kamei, Teruki Kidani, Haruyasu Yamamoto, Yoshiaki Norimatsu, Hiroshi Masuno

**Affiliations:** 1 Department of Bone and Joint Surgery, Ehime University Graduate School of Medicine, Toon, Ehime 791-0295, Japan; 2 Department of Medical Technology, Faculty of Health Sciences, Ehime Prefectural University of Health Sciences, Takooda, Tobe-cho, Iyo-gun, Ehime 791-2101, Japan

## Abstract

**Background:**

Osteosarcoma often develops micrometastases in the lung prior to diagnosis, causing a fatal outcome. Therefore, the prevention of pulmonary metastases is critical for the improvement of the prognosis of patients with osteosarcoma. The purpose of this study was to investigate whether troglitazone (TGZ) is considered as possible therapeutics in the treatment of growth and metastasis of osteosarcoma.

**Methods:**

LM8 cells were treated for 3 days with various concentrations of TGZ. The effect of TGZ on cell proliferation was determined by DNA measurement in the cultures and 5-bromo-2'-deoxyuridine incorporation study. The assay of cell invasion and motility was performed using either the Matrigel-coated cell culture inserts or the uncoated cell culture inserts in the invasion chambers. The effect of TGZ on Akt signaling was assessed by Western blot analysis of Akt and p-Akt. The effects of oral administration of either TGZ (TGZ group) or ethanol (control group) on the growth of primary tumor and the development of pulmonary metastasis were examined in nude mice implanted with LM8 cells on their backs. The expression and activity of matrix metalloproteinase 2 (MMP-2) within the tumor were determined by immunohistochemistry and zymography. The microvessel density (MVD) within the tumor was determined by immunohistochemistry for CD34.

**Results:**

TGZ dose-dependently inhibits cell proliferation. TGZ-treated cells were less invasive and less motile than untreated cells. The activity of MMP-2 secreted by TGZ-treated cells was lower than that secreted by untreated cells. TGZ decreased the level of p-Akt. The primary tumor mass was smaller in the TGZ group than in the control group. The TGZ group had less metastatic tumors in the lung compared with the control group. The expression and activity of MMP-2 within the tumor of the TGZ group were lower than those of the control group. The MVD within the tumor of the TGZ group was lower than that of the control group.

**Conclusions:**

Inhibition of Akt signaling by TGZ may decrease the secretion of MMP-2, resulting in the decrease of invasiveness and motility in LM8 cells. Treatment of tumor-bearing mice with TGZ decreases the expression and activity of MMP-2 within the tumor, and inhibits primary tumor growth and pulmonary metastasis development. TGZ may offer a new approach in chemotherapy for osteosarcoma.

## Background

The peroxisome proliferator-activated receptor γ (PPARγ), which is a member of the nuclear receptor superfamily [1], is expressed mainly in adipose tissue and functions as a key molecule in adipogenesis [2,3]. In addition to adipose tissue, the presence of PPARγ has been demonstrated in a wide variety of tumor cells, which include osteosarcoma cell lines (MG-63, G292, SAOS, U2OS) [4,5], human breast cancer cell lines (MCF-7, MDA-MB-231) [6,7], bladder and prostate cancer cell lines (TSU-Pr1, DU145) [7,8] and a murine mammary tumor cell line (LMM3) [9].

Thiazolidinediones, which include troglitazone (TGZ), rosiglitazone (RGZ), and ciglitazone, are synthetic PPARγ ligands [10]. Several lines of evidence have shown that PPARγ ligands affect tumor growth and apoptosis *in vitro *and *in vivo *[4-9]. Haydon et al. [4] reported that TGZ at 20-100 μM inhibits the growth of MG-63 cells. However, there is a contradictory report that demonstrated that TGZ at 5 and 50 μM does not affect the proliferation of MG-63 cells but increases cell survival, resulting in increased osteosarcoma cell growth [5]. Magenta et al. [9] reported that RGZ at either 1 or 100 μM reduces the viability of LMM3 cells *in vitro*; however, administration of 100 μM RGZ (in the drinking water) to mice implanted s.c. with LMM3 cells in their flanks does not affect primary tumor growth. Thus, the role of PPARγ ligands in tumor biology is controversial.

Osteosarcoma is the most common malignant musculoskeletal tumor and occurs mainly in the metaphyseal region of the long bones of young people. Osteosarcoma expands the cortex of the bone, later erupts through the cortex into the soft tissues, and often leads to the development of micrometastases in the lung prior to diagnosis. Multimodality treatment consisting of aggressive adjuvant chemotherapy and wide tumor excision improves the prognosis of this disease; however, the development of metastatic lesions often causes a fatal outcome [11]. Therefore, in addition to the surgical removal of the primary tumor, the prevention of pulmonary metastases during the early stage of tumor development is critical for the improvement of the prognosis of patients with osteosarcoma.

A number of factors, which include matrix metalloproteinase 2 (MMP-2) [12,13] and vascular endothelial growth factor (VEGF) [14,15], are involved in tumor metastasis. The LM8 cell line, which was established from Dunn murine osteosarcoma, expresses MMP-2 and VEGF, possesses an extremely high metastatic potency, and has been used as an excellent tool for the study of inhibitory agents against pulmonary metastasis [16]. Our recent study showed that topical administration of ketoprofen, which is a nonsteroidal anti-inflammatory drug, to nude mice implanted s.c. with LM8 cells on their backs inhibited tumor growth at the primary site, decreased the expression of MMP-2 and VEGF within the tumor, and decreased the pulmonary metastatic incidence [17]. In the present study, we performed *in vitro *and *in vivo *experiments to analyze the effects of TGZ on tumor growth and pulmonary metastasis development of LM 8 cells, as well as on the expression of MMP-2 and VEGF. We also examined whether TGZ affects Akt signaling in LM8 cells, because phosphatidylinositol 3-kinase (PI3K)-Akt signaling plays an important role in pulmonary metastatic nodule formation in mice implanted s.c. with LM8 cells on their backs [18]. As, to our knowledge, there are no reports on the expression of PPARγ in LM8 cells, we also examined whether LM8 cells express PPARγ using immunofluorescence staining and Western blot analysis.

## Methods

### Reagents and antibodies

TGZ (generously donated by Daiichi-Sankyo Co., Ltd., Tokyo, Japan) was dissolved in DMSO for the *in vitro *study and in 100% ethanol for the *in vivo *study. GW9662 (Sigma-Aldrich, St. Louis, MO, USA) was dissolved in DMSO. For immunohistochemical staining, the following antibodies were diluted with phosphate-buffered saline (PBS): mouse monoclonal antibodies to proliferating cell nuclear antigen (PCNA) and VEGF (Santa Cruz Biotechnology, Santa Cruz, CA, USA) were diluted to 1:100 and 1:50, respectively, and mouse monoclonal antibodies to MMP-2 and CD34 (Novocastra Laboratories Ltd., UK) were diluted to 1:40 and 1:25, respectively. For immunofluorescence staining, the following antibodies were diluted with PBS containing 1% bovine serum albumin (BSA): a mouse monoclonal anti-5-bromo-2'-deoxyuridine (BrdU; Dako Japan, Inc., Tokyo, Japan) and a rabbit polyclonal antibody to PPARγ (Santa Cruz Biotechnology) were diluted to 1:10 and 1:15, respectively, and a fluorescein isothiocyanate (FITC)-labeled anti-mouse IgG (Zymed Laboratories Inc., San Francisco, CA, USA) and an FITC-labeled anti-rabbit IgG (Santa Cruz Biotechnology) were diluted to 1:20. For Western blot analysis, the primary antibodies (a mouse monoclonal antibody to Akt1, rabbit polyclonal antibodies to p-Akt1/2/3, PPARγ, and β-actin; Santa Cruz Biotechnology) were diluted to 1:500-1,000 and a horseradish peroxidase (HRP)-conjugated secondary antibody (GE Healthcare UK Ltd., Buckinghamshire, UK) were diluted to 1:25,000.

### Cell culture

LM8 cells (Riken BRC Cell Bank, Ibaraki, Japan) at a concentration of 1.25 × 10^3 ^cells/cm^2 ^were seeded on a 35 mm plate in the culture medium, which contained 10% fetal bovine serum (FBS), 100 units/ml penicillin, and 100 μg/ml streptomycin in Dulbecco's modified Eagle's medium (DMEM). After 24 h of seeding, the medium was replaced with culture medium containing TGZ at the indicated concentrations. Subconfluent cells were incubated for 24-72 h, harvested in 0.3 ml of solution A (10 mM Tris, 0.1% Triton X-100, pH 7.5), sonicated briefly at 0°C, and centrifuged. An aliquot of the supernatant was used to measure DNA fluorometrically by the method of Hinegardner [19].

### Hematoxylin-eosin (HE) staining and immunofluorescence staining

To examine cell morphology, LM8 cells (1.19 × 10^3 ^cells/cm^2^) were seeded on a 2-well chamber slide (Nalge Nunc International, Osaka, Japan). After 24 h of seeding, subconfluent cells were treated with or without 50 μM TGZ for 3 days, fixed in 70% ethanol for 30 min, incubated in 100% ethanol for 10 min, and stained with HE. The appearance of cells was viewed and photographed under a light microscope. The ratio of spindle-shaped cells to total cells in a field was estimated, with the exception of multilayered cells.

The labeling of DNA with BrdU (Wako Pure Chemicals Co., Osaka, Japan) was performed as described previously [20]. Briefly, after 24 h of seeding, subconfluent cells were treated with or without 50 μM TGZ for 3 days on a 2-well chamber slide. The cells were incubated with 30 μM BrdU during the last 2 h of the 3-day treatment period, fixed with ethanol as described above, treated with 1.5 N HCl for 30 min, and treated with 0.5% Tween 20 for 5 min. Thereafter, the cells were incubated for 1 h with a mouse monoclonal anti-BrdU antibody, followed by a 1 h incubation with an FITC-labeled anti-mouse IgG in the dark, and were then mounted in fluorescence mounting medium (Dako Japan, Inc.). The BrdU-positive cells were observed under fluorescence microscopy and photographed. The BrdU-labeling index was calculated by dividing the number of BrdU-positive cells by the number of total cells in a field.

For immunofluorescence staining of PPARγ, after 24 h of seeding, subconfluent cells were treated with or without 50 μM TGZ for 3 days on a 2-well chamber slide. The cells were fixed with ethanol and treated with 0.5% Tween 20, as described above. Thereafter, the cells were incubated for 1 h with a rabbit polyclonal antibody to PPARγ, followed by a 1 h incubation with an FITC-labeled anti-rabbit IgG in the dark, and were then mounted in fluorescence mounting medium.

### Cell invasion and motility assay

The BD BioCoat Matrigel™ invasion chambers with polyethylene terephthalate-filters coated with matrigel basement membrane matrix (6 wells, 8 μm pore size; BD Biosciences, Franklin Lake, NJ) were re-hydrated just before the assay using FBS-free DMEM according to the manufacturer's instructions. The chambers were assembled using freshly prepared matrigel-coated filters and DMEM containing 10% FBS as a chemoattractant in the lower compartment. Subconfluent LM8 cells, which had been previously treated with or without 50 μM TGZ for 3 days, were harvested by trypsinization, and suspended in DMEM containing 0.1% BSA. The cells (at a concentration of 5 × 10^5 ^cells/2 ml) were added to the invasion chamber containing a matrigel-coated filter. The assembled chambers were incubated for 48 h at 37°C. At the end of the incubation, nonmigrating cells, which remained on the upper surface of the filter, were completely removed by wiping with a cotton swab. The cells on the bottom surface of the filter were fixed with 100% ethanol for 30 sec and stained with toluidine blue for 10 min. The filters were washed with ethanol and dried. The dye was dissolved with 10% acetic acid and quantitated by measuring the absorbance at 590 nm.

Cell motility was determined as described for the invasion assay, with two modifications: the 12-well cell culture insert with a polyethylene terephthalate-filter (8 μm pore size) was not coated with matrigel and the assembled chambers containing the cells at a concentration of 2.5 × 10^5 ^cells/ml were incubated for 24 h at 37°C.

### Assay of MMP-2 by gelatin zymography

Subconfluent LM8 cells were treated with or without 50 μM TGZ for 2 days. The cells were washed 3 times with PBS and incubated with FBS-free DMEM for 1 h. The medium was then replaced with FBS-free DMEM with or without 50 μM TGZ, and the cells were incubated for an additional 24 h. The conditioned media of these cultures were filtered through 0.2 μm filters. The same amount of medium protein (8.7 μg/lane) was resolved on sodium dodecyl sulfate-polyacrylamide gel electrophoresis (SDS-PAGE, 7.5% acrylamide gel) containing 0.1% gelatin under nonreducing conditions. The gels were then incubated in 20 mM Tris buffer (pH 8.0) containing 2.5% Triton X-100 for 30 min at room temperature, which was followed by incubation for 24 h at 37°C in 20 mM Tris buffer (pH 8.0) containing 1 μM ZnCl_2 _and 10 mM CaCl_2_. The gels were stained with PAGE blue (Cosmo Bio Co., Ltd., Tokyo, Japan). The activity of MMP-2 was identified as a clear band in a blue background. Relative densitometric units were determined using the analysis software, Diversity Database™ (v. 1.1, Toyobo Co., Ltd., Osaka, Japan).

In another series of experiments, a 100 mg tumor sample was homogenized in 2 ml of solution A using a Teflon-glass homogenizer at 0°C and centrifuged. The same amount of supernatant protein (10 μg/lane) was separated by gelatin-SDS-PAGE and the activity of MMP-2 was assayed as described above.

### Western blot analysis

Subconfluent LM 8 cells were treated for 3 days with TGZ at the indicated concentrations, harvested in 0.3 ml of solution A containing a protease inhibitor cocktail (1:100 dilution; Calbiochem-Novabiochem Co., La Jolla, CA), sonicated briefly at 0°C, and centrifuged. The same amount of supernatant protein (15 μg/lane) was separated by SDS-PAGE (10% acrylamide gel) and transferred onto a PVDF membrane, as described previously [21]. The membrane was incubated for 1 h with the primary antibody and was then incubated for 1 h with an HRP-conjugated secondary antibody. Blots were visualized using the ECL Advance Western Blotting Detection Kit (GE Healthcare UK Ltd.), according to the manufacturer's instructions. The membrane was exposed to an X-ray film with an intensifying screen. Relative densitometric units were determined as described above.

### Tumor implantation and animal treatment

LM8 cells (1.05 × 10^6 ^cells/0.3 ml of PBS) were s.c. implanted in the backs of 4-week-old male BALB/cA Jcl-*nu *nude mice (Clea Japan, Inc., Tokyo, Japan) under ether anesthesia. Two mice were housed in a standard polypropylene mouse cage in a 12 h light-dark cycle (lights on at 7 am) and were allowed free access to laboratory chow and water. Tumor-bearing mice were randomly divided into 2 groups (14 mice/group). One group, which was termed the TGZ group, was provided with drinking water containing 100 μM TGZ in 0.5% ethanol, whereas the second group, which was termed the control group, was given drinking water containing 0.5% ethanol. The drinking water was changed every 2-3 days. After 21 days of treatment, the animals were sacrificed under ether anesthesia. The tumors and lungs were excised, weighed, fixed in 10% formalin, and embedded in paraffin. The lung sections (4 μm) with the largest tissue area were stained with HE and evaluated microscopically to confirm the presence of metastatic tumors. The proportional area of pulmonary metastasis in the lung sections was calculated by dividing the sum of the areas of pulmonary metastasis by the entire area of lung tissue.

All animals were treated humanely, and care was taken to alleviate suffering. The experimental protocols were reviewed and approved by the local Animal Ethics Committees at the Ehime University Graduate School of Medicine (approval no. 05-NU-65-14) and at the Ehime Prefectural University of Health Sciences (approval no. 51), Ehime, Japan.

### Immunohistochemical studies

The formalin-fixed, paraffin-embedded tumor sections (4 μm) were deparaffinized and rehydrated, which were followed by antigen retrieval using autoclaving (15 min at 121°C) in 10 mM citrate buffer (pH 6.0) for PCNA and VEGF, and in 1 mM EDTA solution (pH 8.0) for MMP-2. For the detection of CD34, sections were treated for 15 min with proteinase K (20 μg/ml) at room temperature. The sections were incubated overnight at 4°C with a primary antibody and were then incubated for 1 h with HRP-conjugated ENVISION+ (Dako Japan, Inc.), as described previously [17]. Positive cells were visualized by adding diaminobenthidine to the sections. The nuclei were counterstained with hematoxylin. The tissue sections were observed under high-power magnification (400 ×). Different microscopic fields were photographed per tumor and the positive cells present in 500-800 cells per photograph were counted. The labeling index was calculated by dividing the number of positive cells by the number of total cells.

### Statistical analyses

Significant differences among 3-6 independent groups were evaluated using one-way ANOVA and subsequent comparisons were performed using the Tukey-Kramer test. Significant differences between 2 independent groups were analyzed using Student's *t*-test. Pearson's *r *was used to calculate the correlation between body weight, tumor weight, and proportional area. For all statistical analyses, the criterion for significance was p < 0.05. All values were expressed as the means ± SE.

## Results

### Effect of TGZ on cell morphology and PPARγ expression

**Figure 1:**
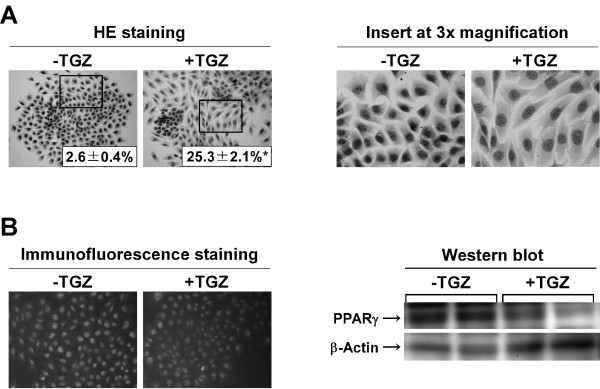
**Effect of TGZ on cell morphology and PPARγ expression**. (A) Subconfluent LM8 cells were treated with or without 50 μM TGZ for 3 days, fixed with ethanol, and stained with HE. The values given in the photographs represent the ratio of the number of spindle-shaped cells to the number of total cells and are the means ± SE for five fields. Magnification: ×200. (B) Subconfluent LM8 cells were treated with or without 50 μM TGZ for 3 days, and the expression of PPARγ was examined by immunofluorescence staining (magnification: ×200) and Western blot analysis.

Since fillopodial and lamellipodial structures surrounding the cell surface of LM8 cells play a pivotal role in cell motility [16], we first studied the effect of TGZ on cell morphology. Almost all cells in the untreated cultures (i.e., TGZ was absent during the 3-day treatment period) were cuboidal in shape (Figure 1A). The TGZ-treated cultures, in which 50 μM TGZ was present during the same period, contained two morphologically different cell types; one was cuboidal in shape and the other was spindle shaped, which was characterized by cell spreading and a larger cell surface area compared with the cuboidal-shaped cells, as indicated by the magnified inserts (Figure 1A, right panel). Approximately 25% of cells in the TGZ-treated cultures were spindle shaped. These findings suggest that TGZ-treated cells may display lower motile activity than untreated cells.

TGZ is a PPARγ ligand [1]. To date, however, it remains unknown whether LM8 cells synthesize PPARγ. Therefore, we examined the expression of PPARγ in LM8 cells using immunofluorescence staining and Western blot analysis (Figure 1B). Positive PPARγ immunofluorescence staining in the nucleus was observed in both the untreated and TGZ-treated cultures; however, the intensity of the fluorescent signal was weaker in the TGZ-treated cultures than in the untreated cultures. Western blot analysis also revealed lower levels of the PPARγ protein in the TGZ-treated cultures compared with the untreated cultures. These results suggest that TGZ inhibits the expression of PPARγ in LM8 cells.

### Effect of TGZ on cell proliferation

**Figure 2:**
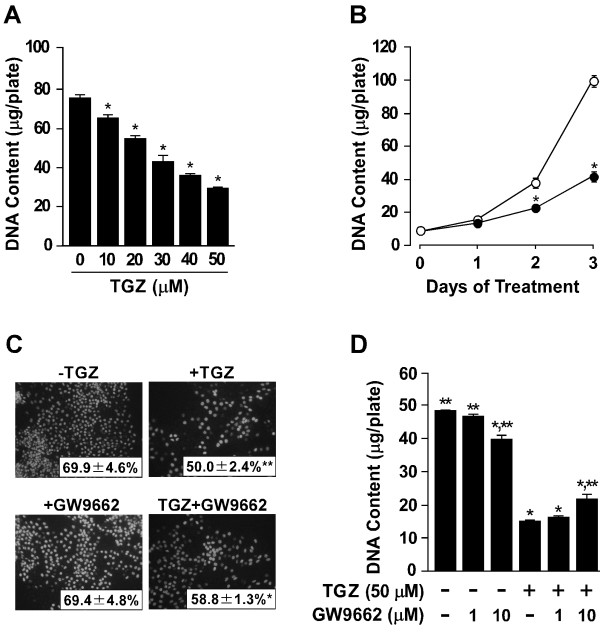
**Effect of TGZ on cell proliferation**. (A) Subconfluent LM8 cells were treated for 3 days with 0-50 μM TGZ, and the DNA content of the cultures was measured. Values given are the means ± SE for four plates. *p < 0.01 (compared with the untreated cultures). (B) Subconfluent LM8 cells were treated with (filled circle) or without (open circle) 50 μM TGZ, and the DNA content of the cultures at the indicated intervals was measured. Values given are the means ± SE for four plates. *p < 0.01 (compared with the untreated cultures on the corresponding day). (C) Subconfluent LM8 cells were treated for 3 days with or without 50 μM TGZ in the absence or presence of 10 μM GW9662 and were incubated with 30 μM BrdU during the last 2 hr of the 3-day treatment period. The fixed cells were incubated for 1 h with a mouse monoclonal anti-BrdU antibody followed by a 1 h incubation with an FITC-labeled anti-mouse IgG. The values given in the photographs represent the BrdU-labeling index and are the means ± SE for five determinations. *p < 0.05, **p < 0.01 (compared with the untreated cultures). Magnification: ×100. (D) Subconfluent LM8 cells were treated for 3 days with the indicated additive, and the DNA content of the cultures was measured. Values given are the means ± SE for four plates. *p < 0.01 (compared with the untreated cultures). **p < 0.01 (compared with the cultures treated with TGZ alone).

Next, we examined the effect of TGZ on cell proliferation. Subconfluent LM8 cells were treated for 3 days with TGZ at the indicated concentrations, and the DNA content of the cultures was measured (Figure 2A). TGZ decreased the DNA content of the cultures in a dose-dependent manner. The presence of 10 μM TGZ caused a significant decrease in the DNA content. TGZ at 50 μM decreased the DNA content by 61%. Figure 2B shows the time course of the TGZ-induced changes in DNA content. In both the untreated and TGZ-treated cultures, the DNA content increased during the 3-day treatment period. On day 1, there was no difference in the DNA content between the two cultures. On days 2 and 3, the DNA content of the TGZ-treated cultures was significantly lower than that of the untreated cultures. These results suggest that TGZ may suppress cell proliferation. To confirm this, cells were incubated with BrdU during the last 2 h of the 3-day treatment period to label DNA synthesis (Figure 2C). In both the untreated and TGZ-treated cultures, we observed positive BrdU immunofluorescence staining in the nucleus. The BrdU-labeling index of the TGZ-treated cultures was significantly lower than that of the untreated cultures. Thus, TGZ seems to inhibit DNA replication.

### Effect of GW9662 on the TGZ-induced decrease in cell proliferation

To evaluate whether TGZ acted via PPARγ, subconfluent LM8 cells were treated for 3 days with or without 50 μM TGZ in the absence or presence of the PPARγ antagonist GW9662 at 1 or 10 μM. The DNA content of the cultures treated with a combination of TGZ and GW9662 at 10 μM, but not at 1 μM, was significantly higher than that of the cultures treated with TGZ alone, but was still significantly lower than that of the untreated cultures (Figure 2D). Thus, GW9662 failed to block completely the TGZ-induced decrease in DNA content. Similarly, the BrdU-labeling index of the cultures treated with a combination of TGZ and GW9662 at 10 μM tended to be higher than that of the cultures treated with TGZ alone, and was significantly lower than that of the untreated cultures (Figure 2C). Treatment with GW9662 at 10 μM, but not at 1 μM, decreased the DNA content by 18% compared with the untreated cultures but did not affect the BrdU-labeling index, which suggests that 10 μM GW9662 *per se *may impair the survival of LM8 cells. Schaefer et al. [22] reported that GW9662 at 10-100 μM induces apoptosis in cultures of the murine colorectal carcinoma cell line HT-29.

### Effect of TGZ on cell invasion, cell motility, and MMP-2 secretion

**Figure 3:**
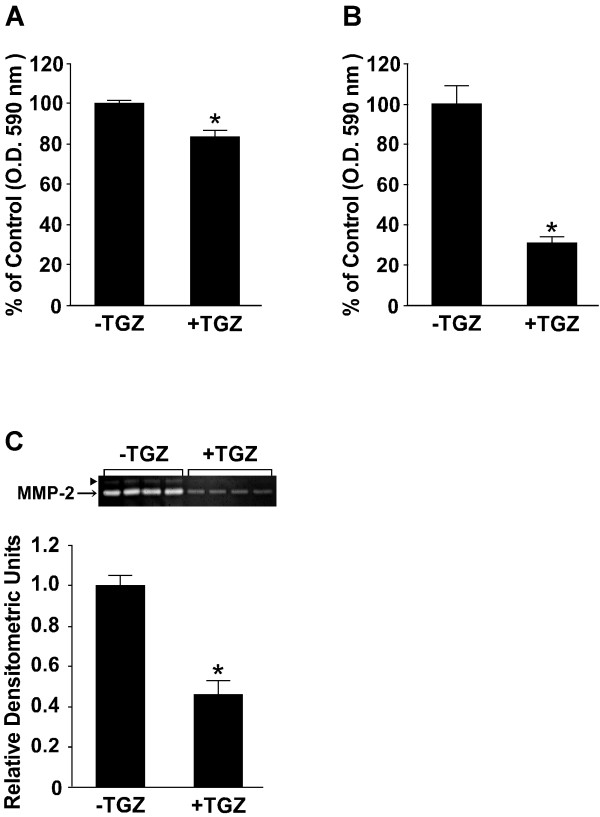
**Effect of TGZ on cell invasion (A), cell motility (B), and MMP-2 secretion (C)**. (A and B) Subconfluent LM8 cells were treated for 3 days with or without 50 μM TGZ and were harvested by trypsinization. Cell invasion and motility assays were performed using either filters coated with matrigel basement membrane matrix (A) or uncoated filters (B). Values given are the means ± SE for six filters. *p < 0.01 (compared with the untreated cultures). (C) Subconfluent LM8 cells were treated for 3 days with or without 50 μM TGZ and the activity of MMP-2 secreted into the medium during the last 24 h of the 3-day treatment was assayed using gelatin zymography. The upper panel shows a gelatine zymogram. Arrowhead in the upper panel shows the proform of MMP-2. Values given in the lower panel are the means ± SE for four plates. *p < 0.01 (compared with the untreated cultures).

Subconfluent LM8 cells were treated for 3 days with or without 50 μM TGZ and were harvested by trypsinization to be used for cell invasion and motility assays. When experiments were performed using filters coated with reconstructed basement membrane, the absorbance of the dye extracted from the TGZ-treated cells was 83% of that extracted from untreated cells (p < 0.01) (Figure 3A). This indicates that TGZ-treated cells were less invasive than untreated cells.

When experiments were performed using uncoated filters, the absorbance of the dye extracted from the TGZ-treated cells was 31% of that extracted from untreated cells (p < 0.01) (Figure 3B). This indicates that TGZ-treated cells were less motile than untreated cells. This low motile activity of TGZ-treated cells may result from the above-mentioned TGZ-induced cell morphological changes.

The activity of MMP-2 secreted into conditioned media during the last 24 h of the 3-day treatment period was assayed by gelatin zymography (Figure 3C). The medium protein concentration of the TGZ-treated cultures was higher than that of the untreated cultures [untreated cultures (n = 4), 1.01 ± 0.02 mg/plate; TGZ-treated cultures (n = 4), 1.09 ± 0.01 mg/plate; p < 0.01]. LM8 cells in both the untreated and TGZ-treated cultures secreted MMP-2; however, the activity of MMP-2 secreted from TGZ-treated cells was 46% of that secreted from untreated cells (p < 0.01).

### Effect of TGZ on Akt signaling

**Figure 4:**
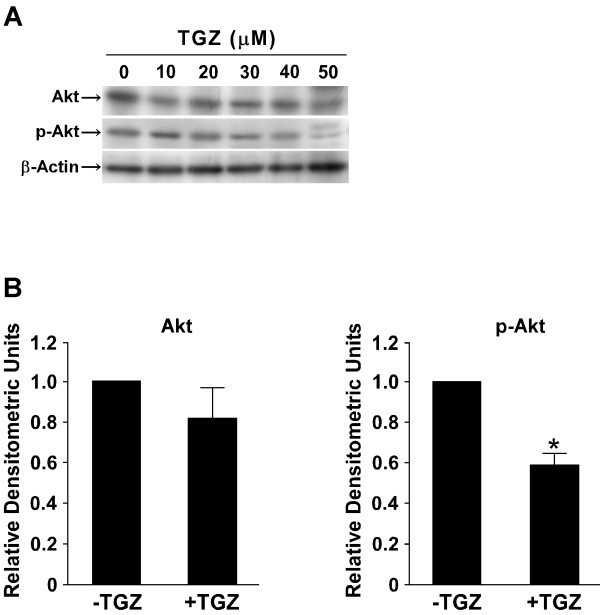
**Effect of TGZ on the levels of Akt and p-Akt**. (A) Subconfluent LM8 cells were treated for 3 days with TGZ at the indicated concentrations. The proteins (15 μg of protein/lane) in the supernatant of cell homogenates were separated by SDS-PAGE and the expression of Akt and p-Akt was analyzed by Western blot. (B) Subconfluent LM8 cells were treated for 3 days with or without 50 μM TGZ. The expression of Akt and p-Akt was analyzed by Western blot, and relative densitometric units were determined using the analysis software. Values given are the means ± SE for five plates. *p < 0.01 (compared with the untreated cultures).

Inhibition of Akt signaling in LM8 cells results in the suppression of the secretion of MMP-2 and the *in vitro *invasiveness and motility [18]. Therefore, subconfluent LM8 cells were treated for 3 days with TGZ at the indicated concentrations and the levels of Akt and p-Akt were analyzed by Western blot analysis. TGZ decreased the level of p-Akt in a dose-dependent manner (Figure 4A). TGZ at 50 μM decreased the level of p-Akt by 58% compared with the untreated cultures (p < 0.01) (Figure 4B). The level of Akt in the cultures treated with 50 μM TGZ was lower than that in the untreated cultures, but this difference was not significant.

### Effect of TGZ on tumor growth and pulmonary metastasis development in mice implanted with LM8 cells

**Figure 5:**
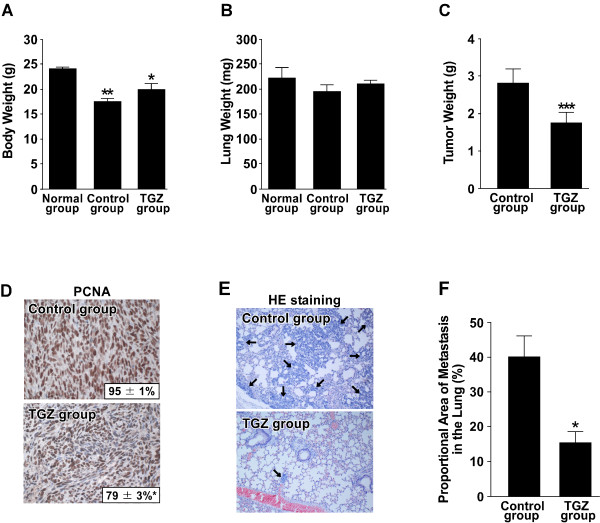
**Effect of TGZ on tumor growth and pulmonary metastasis**. (A-C) After 21 days of TGZ treatment, the body weight (A), lung weight (B), and tumor weight (C) were measured. Body weight was measured after the excision of the tumor. Values given are the means ± SE for six mice in the normal group, thirteen mice in the control group, and fourteen mice in the TGZ group. *p < 0.05, **p < 0.01 (compared with the normal group). ***p < 0.05 (compared with the control group). (D) Immunohistochemistry for PCNA was performed using the primary tumor sections. The values given in photographs show the PCNA-labeling index and are the means ± SE for five determinations in the control group and ten determinations in the TGZ group. *p < 0.01 (compared with the control group). (E) HE staining of the lung sections was performed. Arrow: metastatic tumors. (F) The proportional area of pulmonary metastasis was calculated by dividing the sum of the areas of lung metastasis by the entire area of lung tissue. Values given are the means ± SE for thirteen mice in the control group and fourteen mice in the TGZ group. Magnification: ×100. *p < 0.01 (compared with the control group).

LM8 cells were s.c. implanted on the backs of nude mice, and either 100 μM TGZ (TGZ group) or 0.5% alcohol alone (control group) was administered in the drinking water to tumor-bearing mice for 3 weeks. In the control group, one of 14 mice died during the experimental period, while the survival rate of the TGZ group was 100%. Progressive weight loss occurred in both the control and TGZ groups compared with age-matched normal nude mice, which were termed the normal group [F(2,30) = 9.736, p < 0.001] (Figure 5A). The mean body weight of the control and TGZ groups was 73% (p < 0.01) and 83% (p < 0.05), respectively, of that of the normal group [23.9 ± 0.4 g (n = 6)]. There was no difference in the mean lung weight among the three groups (Figure 5B).

The tumor mass of the control group (n = 13) was 2.81 ± 0.38 g. In the TGZ group, the mean tumor mass was 62% of that of the control group (p < 0.05) (Figure 5C). When the data (n = 27) of the two tumor-bearing groups were analyzed together, tumor weight correlated negatively with body weight (r = -0.436, p < 0.05).

To examine the effect of TGZ on tumor growth, we performed immunohistochemistry for PCNA, which is expressed in the nuclei of proliferating cells [23-25], using the primary tumor sections (Figure 5D). In the control group, positive PCNA nuclear immunostaining was extensively observed within the tumors, and the mean PCNA-labeling index was 95%. In the TGZ group, there were less PCNA-positive cells compared with the control group, and the mean labeling index was 79% (p < 0.01).

The 13 mice included in the control group had multiple metastatic tumors in the lung (Figure 5E), and the proportional area of pulmonary metastasis was 40 ± 6% (Figure 5F). The 14 mice included in the TGZ group had less metastatic tumors compared with the control group, and the proportional area was 15 ± 3% (p < 0.01). The proportional area correlated positively with primary tumor weight (r = 0.567, p < 0.01) and negatively with body weight (r = -0.446, p < 0.05).

### Effect of TGZ on the expression and activity of MMP-2 within tumors

**Figure 6:**
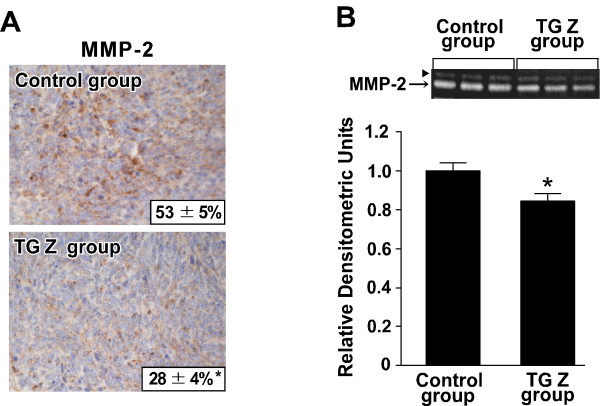
**Effect of TGZ on the expression and activity of MMP-2 within the tumor**. (A) Immunohistochemistry for MMP-2 was performed using the primary tumor sections. The values given in photographs show the MMP-2-labeling index and are the means ± SE for five determinations in the control group and ten determinations in the TGZ group. *p < 0.01 (compared with the control group). (B) The activity of MMP-2 in the supernatant (10 μg of protein/lane) of tumor homogenate was assayed using gelatin zymography. The upper panel shows a representative gelatine zymogram. Arrowhead in the upper panel shows the proform of MMP-2. Values given in the lower panel are the means ± SE for ten tumor specimens in the control group and fourteen tumor specimens in the TGZ group. *p < 0.05 (compared with the control group).

The presence of active MMP-2 and -9 in the tumor results in alterations to the microenvironment that promote tumor invasion and metastasis [26]. LM8 cells express mainly MMP-2 [16]. Therefore, we performed immunohistochemistry for MMP-2 using the primary tumor sections (Figure 6A). In the control group, positive MMP-2 immunostaining was observed in the cytoplasm, and the mean MMP-2-labeling index was 53%. In the TGZ group, tumors contained less MMP-2-positive cells compared with the control group, and the mean labeling index was 28% (p < 0.01).

Tumors were homogenized and centrifuged, and the activity of MMP-2 in the supernatants was measured using gelatin zymography (Figure 6B). The activity of MMP-2 in the TGZ group was 85% of that of the control group (p < 0.05).

### Effect of TGZ on the expression of VEGF and CD34 within tumors

**Figure 7:**
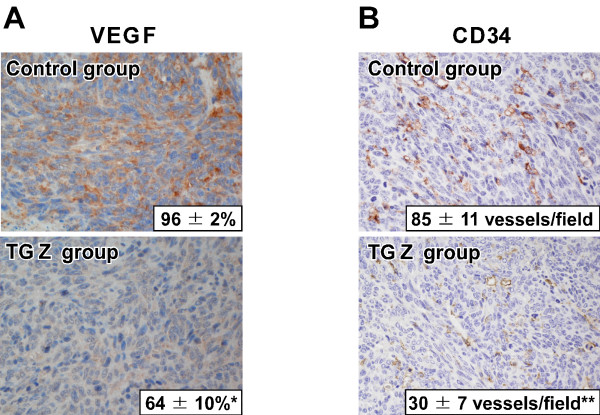
**Effect of TGZ on the expression of VEGF and CD34 within the tumor**. (A) Immunohistochemistry for VEGF was performed using the primary tumor sections. The values given in photographs show the VEGF-labeling index and are the means ± SE for five determinations in the control group and ten determinations in the TGZ group. *p < 0.05 (compared with the control group). Magnification: ×400. (B) Immunohistochemistry for CD34 was performed using the primary tumor sections. MVD was assessed in the tumor sections stained with the anti-CD34 antibody The values given in photographs show the MVD and are the means ± SE for five determinations in the control group and ten determinations in the TGZ group. **p < 0.01 (compared with the control group). Magnification: ×400.

The tumors derived from high VEGF-expressing osteosarcoma cells grow more rapidly and are more likely to metastasize to the lung than the tumors derived from low VEGF-expressing cells [15]. Therefore, we performed immunohistochemistry for VEGF using the primary tumor sections. In the control group, positive VEGF immunostaining was extensively observed in the cytoplasm, and the mean VEGF-labeling index was 96% (Figure 7A). In the TGZ group, there were less VEGF-positive cells compared with the control group, and the mean labeling index was 64% (p < 0.05).

Local vessel formation is associated with the growth and metastasis of tumor [17,25,27]. Therefore, we assessed microvessel density (MVD) in the tumor sections stained with the anti-CD34 antibody (Figure 7B). The mean MVD of the control group was 85 vessels/field. The mean MVD of the TGZ group was 30 vessels/field, which was lower than that of the control group (p < 0.01).

## Discussion

The purpose of this study was to investigate whether TGZ is considered as possible further therapeutics in the treatment of growth and metastasis of osteosarcoma. To explore this, we examined the *in vitro *and *in vivo *effects of TGZ on tumor growth and pulmonary metastasis development of LM8 cells, which grow at high proliferative rate and possess high metastatic potential to the lung [16]. The results of DNA measurement and BrdU incorporation into DNA revealed that TGZ inhibited DNA replication and cell proliferation in cultures of LM8 cells (Figure 2A and 2C). The *in vivo *study showed that the primary tumor mass of the control group reached 16% of body weight, whereas the tumor mass of the TGZ group was 9% of body weight (Figure 5A and 5C). This finding suggests that treatment of tumor-bearing mice with TGZ may inhibit tumor growth at the primary site. To confirm this, we immunohistochemically examined the expression of PCNA within primary tumors, because the level of PCNA in the nucleus begins to increase during the late G1-phase immediately before the onset of DNA synthesis, peaks during the S-phase, then decreases again during the G2 and M phases [23]. Since the mean PCNA positivity within primary tumors was 95% in the control group and 79% in the TGZ group (Figure 5D), this indicates that a great majority of tumor cells in the control group were proliferating actively, whereas 21% of tumor cells in the TGZ group were arrested in the G1/G0-phase. A TUNEL assay failed to show an increase in apoptotic cells within the tumor of the TGZ group compared with the control group (data not shown). Based on these findings, we conclude that treatment of LM8 cells with TGZ increases the number of G1/G0-phase arrested cells, thus resulting in the suppression of tumor growth.

Our results of immunofluorescence staining and Western blot analysis, for the first time, demonstrate that LM8 cells express PPARγ protein in the nucleus and that TGZ decreases the expression of PPARγ protein (Figure 1B). These findings raise the question of whether the TGZ-induced decrease in cell proliferation was mediated by PPARγ, because the PPARγ ligands have antitumor activity against a wide variety of tumors *in vitro *[7]. To explore this, we used the PPARγ antagonist GW9662. GW9662 did not completely reverse the TGZ-induced decrease in the DNA content of the cultures (Figure 2D). Similar trends were observed in the production of BrdU-positive cells (Figure 2C). Based on these findings, we conclude that TGZ may, at least partly, suppress the proliferation of LM8 cells via an alternative mechanism that does not involve PPARγ. Several lines of evidence indicate the presence of PPARγ-independent growth inhibition in some cancer types. For example, TGZ induces cellular acidosis and decreases DNA synthesis in MCF-7 and MDA-MB-231 cells; however, GW9662 is not able to block the decreased DNA synthesis associated with TGZ-induced acidosis [28]. Seargent et al. [29] found that RGZ inhibits the growth of MDA-MB-231 cells and that GW9662 enhances rather than reverses RGZ-induced growth inhibition. Chaffer et al. [8] also reported that TGZ induces PPARγ-independent cell cycle arrest at the G1/G0 phase and suppresses tumor cell growth in prostate and bladder carcinomas.

In the present study, we found that TGZ-treated LM8 cells were less invasive and less motile (Figure 3A and 3B) and exhibited lower secretion of MMP-2 compared with untreated cells (Figure 3C). The several lines of evidence reveal that Akt, which functions downstream of PI3K, is associated with the invasiveness and motility of tumor cells [18,30-32] and the secretion of MMP-2 [18,33,34]. Fukaya et al. [18] reported that inhibition of Akt signaling by either the expression of a dominant-negative form of Akt in LM8 cells or the treatment of LM8 cells with LY294002, which is a potent PI3K inhibitor, decreases the secretion of MMP-2 and suppresses cell invasion and motility [18]. Yang et al. [32] reported that TGZ decreases the level of p-Akt and inhibits cell motility in human ovarian carcinoma cell line, ES-2. Therefore, we examined the effect of TGZ on the activation of Akt by Western blot analysis. Since TGZ decreased the level of p-Akt (Figure 4A and 4B), this indicates that TGZ inhibits Akt signaling in LM8 cells. Taken together, the present findings suggest that inhibition of Akt signaling by TGZ may decrease MMP-2 secretion, thus resulting in the decrease of the invasiveness and motility in LM8 cells. These *in vitro *findings raise the question of whether TGZ inhibits the *in vivo *development of metastasis in the lung, because cell invasion and motility are the critical steps in metastasis.

To explore the above question, TGZ was administered in the drinking water to nude mice implanted s.c. with LM8 cells on the back, and the lung sections were stained with HE to evaluate the presence of metastatic tumors. All mice in the control and TGZ-treated groups possessed metastatic tumors in the lung (Figure 5E); however, the proportional area of pulmonary metastasis was markedly smaller in the TGZ group than in the control group (Figure 5F). Thus, treatment of tumor-bearing mice with TGZ suppresses the development of metastasis in the lung. A simple regression analysis showed that the proportional area decreased with decreasing weight of the primary tumor. These *in vivo *findings suggest that the TGZ-induced inhibition of tumor growth at the primary site causes a delay in the development of pulmonary metastasis. Magenta et al. [9] also found that oral administration of 100 μM RGZ to mice injected i.v. with LMM3 mammary tumor cells decreases the formation of tumor nodules on the surface of the lung. However, they reported that it does not inhibit tumor growth at the primary site in mice implanted s.c. with LMM3 cells in the flank.

The destruction of the extracellular matrix (ECM) is the first critical event for tumor invasion and metastasis. MMPs are capable of digesting various components of the ECM and play an important role by removing physical barriers to invasion [12]. In particular, MMP-2 and -9 degrade EMC macromolecules in the basement membranes and other interstitial connective tissues [13]. The results of immunohistochemistry (Figure 6A) and gelatin zymography (Figure 6B) showed that the tumors of the TGZ group exhibited lower expression and activity of MMP-2 compared with the control group. These findings suggest that the TGZ-induced inhibition of MMP-2 expression within the tumor may prevent the local invasion at the primary site, thus resulting in the suppression of the development of metastasis in the lung.

In addition to MMP-2, VEGF expression and local vessel formation are also associated with the growth and invasion of primary tumor, and the development of metastasis. Kaya et al. [14] reported that VEGF expression in primary osteosarcoma correlates with an increase in MVD within the tumor, the development of pulmonary metastasis, and poor prognosis in patients who undergo aggressive therapy. In rodents, a higher expression of VEGF mRNA in LM8 cells facilitates neovascularization at the site of metastasis, thus resulting in an extremely high metastatic potency after i.v. injection [16]. A great majority of the cells within tumors of the control group were VEGF-positive, while 36% of the cells within tumors of the TGZ group were VEGF-negative (Figure 7A). This finding suggests that treatment with TGZ may inhibit neovascularization within primary tumor. Actually, the results of immunohistochemistry for CD34, which has been used as a reliable endothelial marker for MVD assessment [35], showed that the MVD within tumors of the TGZ group was markedly lower compared with the control group (Figure 7B). Taken together, our *in vivo *findings suggest that administration of TGZ not only decreases the expression of MMP-2 and invasiveness, but also decreases the expression of VEGF and local vessel formation, thus preventing both the growth of primary osteosarcoma and the development of metastasis in the lung.

Several lines of evidence indicate that the PI3K-Akt signaling pathway plays an important role in the regulation of tumor metastasis. Fukaya et al. [18] reported that the lungs of mice inoculated with LM8 cells have multiple tumor nodules, whereas mice inoculated with either dominant-negative Akt-expressing LM8 cells or LY294002-treated LM8 cells have no visible tumor nodule on the surface of the lung. Li et al. [36] reported that the helix-loop-helix transcription factor Id-1 (an inhibitor of differentiation and DNA binding), which is expressed in many types of tumors [36-38], promotes tumorigenicity and metastasis of human esophageal cancer *in vivo *and that LY294002 can attenuate these effects. Teranishi et al. [37] reported that the PI3K-Akt signaling pathway plays an essential role in peritoneal metastasis and that PI3K inhibitors such as wortmannin can be novel modalities to prevent peritoneal metastasis of invasive cancers such as pancreatic cancer. Thus, the PI3K-Akt signaling pathway may be a chemotherapeutic target in tumor treatment including osteosarcoma.

## Conclusions

The *in vitro *and *in vivo *studies reveal that TGZ can inhibit tumor growth and pulmonary metastasis development of LM8. We suggest that TGZ may be used as an efficacious adjuvant chemotherapeutic agent for primary osteosarcoma.

## Competing interests

The authors declare that they have no competing interests. Troglitazone is generously donated by Daiichi-Sankyo Co., Ltd., Tokyo, Japan.

## Authors' contributions

JA performed the bulk of experiments *in vitro *and *in vivo*. SK, TK and YN participated in *in vivo *experiments including immunohistochemical study. KS and HY designed experiments and analyzed data. HM is a project leader, designed experiments, analyzed data, and wrote the manuscript. All authors read and approved the final manuscript.

## Pre-publication history

The pre-publication history for this paper can be accessed here:

http://www.biomedcentral.com/1471-2407/10/51/prepub
